# Customized bioceramic scaffolds and metal meshes for challenging large-size mandibular bone defect regeneration and repair

**DOI:** 10.1093/rb/rbad057

**Published:** 2023-06-07

**Authors:** Bin Zhang, Xiaohong Yin, Feng Zhang, Yirong Hong, Yuesheng Qiu, Xianyan Yang, Yifan Li, Cheng Zhong, Huayong Yang, Zhongru Gou

**Affiliations:** State Key Laboratory of Fluid Power & Mechatronic Systems, Zhejiang University, Hangzhou 310058, China; School of Mechanical Engineering, Zhejiang University, Hangzhou 310058, China; State Key Laboratory of Fluid Power & Mechatronic Systems, Zhejiang University, Hangzhou 310058, China; School of Mechanical Engineering, Zhejiang University, Hangzhou 310058, China; Department of Stomatology, Children’s Hospital, Zhejiang University School of Medicine, Hangzhou 310003, China; State Key Laboratory of Fluid Power & Mechatronic Systems, Zhejiang University, Hangzhou 310058, China; School of Mechanical Engineering, Zhejiang University, Hangzhou 310058, China; Department of Stomatology, Children’s Hospital, Zhejiang University School of Medicine, Hangzhou 310003, China; Bio-Nanomaterials and Regenerative Medicine Research Division, Zhejiang-California International Nanosystem Institute, Zhejiang University, Hangzhou 310058, China; Department of Orthopedics, The First Affiliated Hospital, Zhejiang University School of Medicine, Hangzhou 310003, China; Department of Orthopedics, The First Affiliated Hospital, Zhejiang University School of Medicine, Hangzhou 310003, China; State Key Laboratory of Fluid Power & Mechatronic Systems, Zhejiang University, Hangzhou 310058, China; School of Mechanical Engineering, Zhejiang University, Hangzhou 310058, China; Bio-Nanomaterials and Regenerative Medicine Research Division, Zhejiang-California International Nanosystem Institute, Zhejiang University, Hangzhou 310058, China

**Keywords:** customized design, bioceramic scaffolds, titanium meshes, mandibular bone reconstruction, additive manufacturing

## Abstract

Large-size mandible graft has huge needs in clinic caused by infection, tumor, congenital deformity, bone trauma and so on. However, the reconstruction of large-size mandible defect is challenged due to its complex anatomical structure and large-range bone injury. The design and fabrication of porous implants with large segments and specific shapes matching the native mandible remain a considerable challenge. Herein, the 6% Mg-doped calcium silicate (CSi-Mg6) and β- and α-tricalcium phosphate (β-TCP, α-TCP) bioceramics were fabricated by digital light processing as the porous scaffolds of over 50% in porosity, while the titanium mesh was fabricated by selective laser melting. The mechanical tests showed that the initial flexible/compressive resistance of CSi-Mg6 scaffolds was markedly higher than that of β-TCP and α-TCP scaffolds. Cell experiments showed that these materials all had good biocompatibility, while CSi-Mg6 significantly promoted cell proliferation. In the rabbit critically sized mandible bone defects (∼13 mm in length) filled with porous bioceramic scaffolds, the titanium meshes and titanium nails were acted as fixation and load bearing. The results showed that the defects were kept during the observation period in the blank (control) group; in contrast, the osteogenic capability was significantly enhanced in the CSi-Mg6 and α-TCP groups in comparison with the β-TCP group, and these two groups not only had significantly increased new bone formation but also had thicker trabecular and smaller trabecular spacing. Besides, the CSi-Mg6 and α-TCP groups showed appreciable material biodegradation in the later stage (from 8 to 12 weeks) in comparison with the β-TCP scaffolds while the CSi-Mg6 group showed much outstanding mechanical capacity *in vivo* in the early stage compared to the β-TCP and α-TCP groups. Totally, these findings suggest that the combination of customized strength-strong bioactive CSi-Mg6 scaffolds together with titanium meshes is a promising way for repairing the large-size load-bearing mandible defects.

## Graphical abstract

**Figure:**
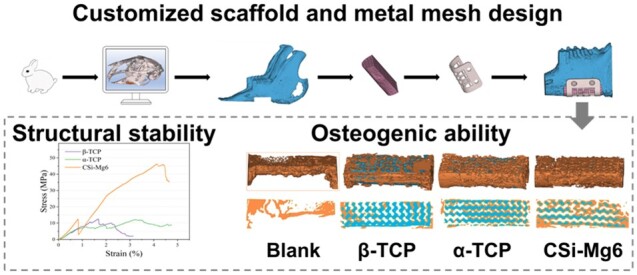

## Introduction

The mandible maintains the irregular curved shape of the maxillofacial part and assists in the realization of physiological functions such as chewing, swallowing and speech [1]. Mandibular defects due to trauma, tumor, infection and other reasons can affect masticatory function and cause changes in the facial shape of the patients, resulting in serious psychological issues [2]. However, large-size defect, that is, defect that is beyond self-repairing capability, can be challenging to repair. Hence, research in synthetic bone grafts for mandible defect repair has boomed [3–5]. It’s believed that the implants need to promote bone tissue ingrowth and repair, which requires the scaffold materials to be biocompatible, osteoconductive, biodegradable [6, 7]. Since the mandible is the load-bearing bone and its longitudinal yield compressive stress value reaches 200 MPa, the scaffolds for challenging large-size mandibular defects repair need both good mechanical properties and stable fixation to the host bone to bear masticatory stress and avoid rupture and movement of the scaffolds [8]. Meanwhile, the scaffolds should have a suitable shape that matches the patient's defect. Hence, the customized scaffold with the excellent osteogenic capability to guide the formation of new bone and stable load-bearing ability to prevent new bone fractures was required.

Recently, many biomaterials have been developed in bone regenerative medicine [9]. The polymeric materials such as polylactic acid, poly(ε-caprolactone) and poly(l-lactic acid) are commonly used due to their controllable mechanical properties and biodegradability [10, 11]. However, the hydrophobic properties of these materials make it difficult for cell adhesion and have insufficient osteogenic ability [12, 13]. Titanium alloys are known for their superior mechanical properties and good processing potential [14]. Unfortunately, the slow degradation of such metal materials hinders their application in the field of bone regeneration. Bioceramics such as Ca-phosphate- and Ca-silicate-based ceramics have been widely used because they usually have excellent osteogenic ability [15–17]. Nevertheless, these bioceramics are brittle and they are not optimal for load-bearing mandible bone critical defect repair.

Nowadays, the hydroxyapatite (HAp), β phase of tricalcium phosphate (β-TCP) and their biphasic mixtures have been widely used in clinic because of their similar chemical composition to bone minerals [18, 19]. The biodegradation rate of HAp is too slow to achieve replacement of the scaffolds by newly formed bone, and then, β-TCP gradually replaces HAp in clinical use [20]. The α phase of tricalcium phosphate (α-TCP) has been shown to be bioactive and possess appreciable osteoconduction, while its poor mechanical properties and rapid degradation rate make it unable to be used in load-bearing bones [21]. The Ca-silicate bioceramics showed high bioactivity and osteoproduction ability [22]. And the superiority of Mg doping has been well established. Mg-doped Baghdadite scaffolds demonstrated favorable mechanical properties, while Mg-substituted calcium silicate (CSi) had stronger promotion effects on cell differentiation than the pure CSi scaffold [23, 24]. In our previous study, the wollastonite bioceramic doped with 6% and 10% magnesium showed higher cell proliferation and differentiation [25]. Therefore, it is reasonable to consider that the combination of such bioceramic scaffold and metal mesh may provide both good osteogenic capacity and reliable mechanical stability.

Moreover, it is agreed that the pore architectures of porous scaffolds also play an important role in osteogenic efficiency, and thus, the manufacturing technology affects the biological performances of scaffolds as well. For instance, the well-interconnected pores can promote osteogenic cell migration and stimulate the bone regeneration while a desirable scaffold shape is needed to match the defect, avoiding ectopic bone formation [26]. In recent years, digital light processing (DLP) has enjoyed great advantages in meeting the individual needs of external morphology and internal microstructures of porous scaffolds because of its high resolution [27–29]. Such advanced stereolithography technique has been developed for the manufacture of bioceramic scaffolds with precise pore size and complete interconnectivity [30], which is capable of creating implants that match bone defects at macro-/micro-scopic levels.

Based on the aforementioned concerns, our aim is to reconstruct the large-size mandibular bone defects by customizing bioceramic scaffolds together with metal meshes. Specifically, the bioceramic scaffold was used to promote new bone ingrowth while the metal mesh was used to offer stable loading capacity to prevent new bone fracture, and a customized shape was used to restore the mandibular morphology. The 6% Mg-doped calcium silicate (CSi-Mg6), β-TCP and α-TCP were used to fabricate the bioceramic scaffolds via DLP while titanium meshes fabricated by selective laser melting (SLM) were acted as fixing supporting. The mechanical and biodegradable analyses were conducted to evaluate the physicochemical properties of the porous scaffolds, and cell experiments were performed to assess the biocompatibility of materials while *in vivo* experiment was executed to study bone repair. Our study showed that the titanium meshes ensured stable mechanical fixation while the biodegradable bioceramic scaffolds offered bone repair potential.

## Materials and methods

### Materials

The reagent-grade inorganic salts such as calcium nitrate tetrahydrate (Ca(NO_3_)_2_ · 4H_2_O), magnesium nitrate hexahydrate (Mg(NO_3_)_2_ · 6H_2_O), sodium metasilicate nonahydrate (Na_2_SiO_3_ · 9H_2_O) and ammonia water were supplied by Sinopharm Reagent Co., China, while the ammonium phosphate ((NH_4_)_2_HPO_4_) was bought from Aladdin Biological Technology Co., China. SP700 photosensitive acrylic resin (Shaoxing Xunshi Technology Co., China) and BYK-111 dispersant (BYK-Chemie GmbH, Germany) were chosen as resin and dispersant for DLP printing. Cell Counting Kit-8 (CCK-8, HY-K0301) was supplied by MedChemExpress, USA. TRITC Phalloidin (FS0381, Shanghai Fushen Biotechnology Co., Ltd, China) and 2-(4-amidinophenyl)-6-indolecarbamidine dihydrochloride (DAPI, Beyotime Biotech, China) were used for F-actin and nuclear staining, respectively. The MC3T3-E1 cell (iCell-m050), minimum essential medium (α-MEM, iCell-0003), fetal bovine serum (FBS, iCell-0500) and penicillin–streptomycin (iCell-15140-122) were purchased from iCell Bioscience, China. The glutaraldehyde solution (G916054) was bought from Macklin Biochemical Co., Ltd, China. The osmic acid was provided by SPI-CHEM. All of these reagents were used without modification.

### Preparation of bioceramic powders

The CSi-Mg6 and TCP powders were both synthesized through a chemical co-precipitation method as described previously [31, 32]. Briefly, Ca(NO_3_)_2_ · 4H2O solution and (NH_4_)_2_HPO_4_ solution (0.5 M) were prepared, and then, Ca(NO_3_)_2_ · 4H2O solution (3.0 l) was slowly dripped into (NH_4_)_2_HPO_4_ solution (2.0 l) under continuous stirring. Diluent ammonia water was used to control the PH between 7.5 and 7.8. As for CSi-Mg6 powders, Ca(NO_3_)_2_ · 4H_2_O solution, Mg(NO_3_)_2_ · 6H_2_O solution and Na_2_SiO_3_ · 9H_2_O solution (0.5M) were prepared, and then, the Ca(NO_3_)_2_ · 4H2O-Mg(NO_3_)_2_ · 6H_2_O mixture solution (6.0 l, 94:6 M ratio) was added into the Na_2_SiO_3_ · 9H_2_O solution (6.0 l) dropwise under continuous stirring. All the resulted suspensions are aged for 36 h and filtered, washed and dried overnight in the oven (80°C). The dried powders were calcined at 800 and 1200°C for 3 hours to obtain β and α phases of TCP, respectively, and the CSi-Mg6 powers were obtained after calcining at 850°C for 3 h. All of the powders were ground in a planetary ball miller for 6 hours. The phase and chemical composition were confirmed by X-ray diffraction (XRD, Rigaku, Japan) and inductively coupled plasma optical emission spectrometer (ICP-OES; Thermo, UK) analysis. The granule morphology of powders was observed by SEM images (SU8010, Hitachi, Japan) and energy-dispersive spectroscopy (EDS, SU8010, Hitachi, Japan) was conducted to show the distribution of Mg elements in CSi-Mg6 powders.

### Design of bioceramic scaffold and metal mesh

Previous studies have reported that more tissue ingrowth occurred in areas with higher porosity after implantation [33]. However, an increase in porosity inevitably resulted in a decrease in mechanical strength [34]. Hence, the porous scaffold with a porosity of over 50% was designed. Since the pore structure was shown to influence the repair of bone, and the triply-periodic minimal surface (TPMS)-based gyroid pore structure achieved ideal new bone formation in the studies while it could be parameterized, the gyroid structure was selected [18, 19, 35]. To prevent soft tissue invasion, the sparse round holes of 300 μm on the surface of the ceramic scaffold in contact with the soft tissue were designed. We deliberately enlarged the round hole size to 300 μm, thus enabling the fabrication of pores sizing 100–200 μm. Otherwise, these pores could not be fabricated because the uncured slurry in the pores could not be cleaned. To prevent the bioceramic scaffold from breaking or moving, a metal mesh that matches the scaffold was designed for temporarily-used osteosynthesis fixation.

**Figure 1.**
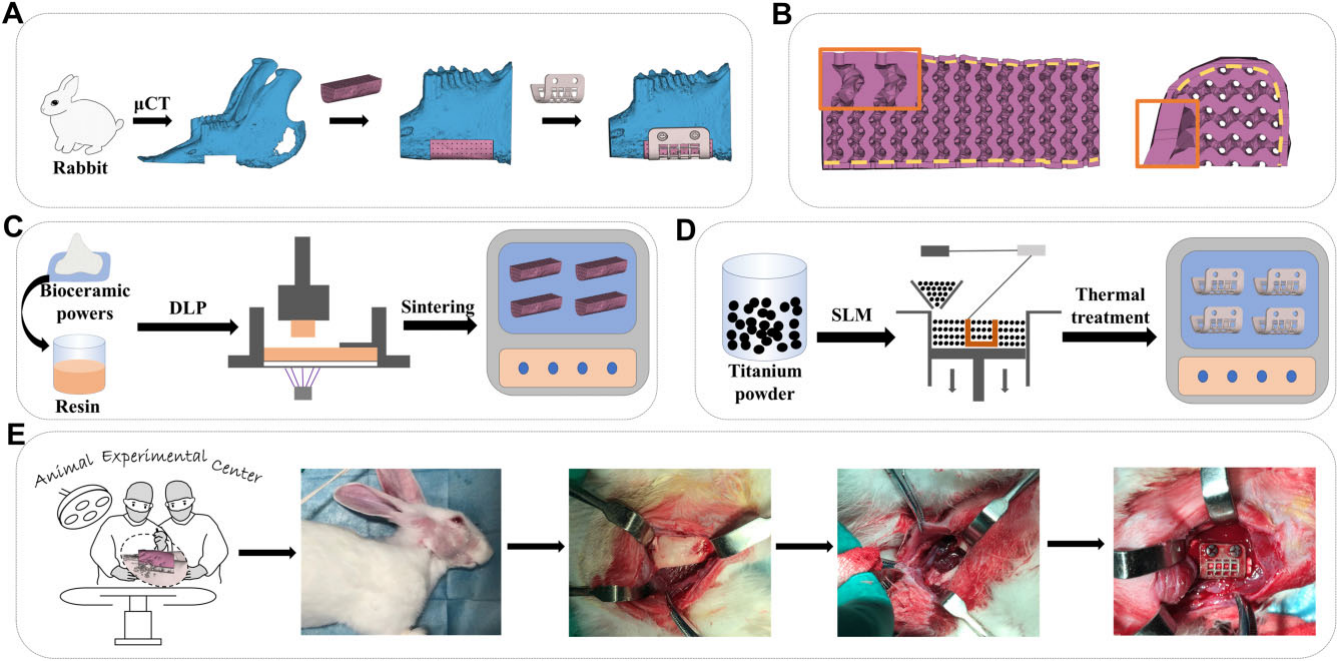
Schematic illustration of the design, preparation, and implantation of the porous implants for mandible repair. (**A**) Design process of personalized bioceramic scaffolds and metal meshes. (**B**) Images of the porous scaffold model. The gold dotted lines marked the boundary of layer 1 and layer 2, and the orange boxes showed the enlarged details. (**C**) Preparation of the bioceramic scaffolds. (**D**) Printing of the metal meshes. (**E**) Surgical procedure of animal experiment.

The models of bioceramic scaffolds and metal meshes were designed as shown in Fig. 1A. First, rabbits anesthetized by 3% sodium phenobarbital at 1.0 ml/kg were scanned using the CT scanner (U-CT, MILabs, Netherlands), and the 3D mandible models were reconstructed based on CT data. The defect model was designed in Magics 24.0 (Materialise, Belgium) according to the defect range (distal to the mandibular first premolar to proximal to the masseter: 13.0 mm  × 5.9 mm  × 4.0 mm). For convenience, a single-period gyroid cell was prepared. And the surface description of the gyroid model with 50% porosity is *Φ_G_* = cos(*ax*)sin(*by*) + cos(*by*)sin(*cz*) + cos(*cz*)sin(*ax*) + *f*(*x*, *y*, *z*) where *a*, *b* and *c* are constants related to pore size and *f*(*x*, *y*, *z*) is used to define the shape and size of the model. Meanwhile, a cylinder (Ø 0.3 mm  × 1 mm) was prepared. Subsequently, the defect model was segmented into an outer soft tissue contact layer (hereinafter referred to as shell; thickness: 0.3 mm) and an inner bone layer (hereinafter referred to as inner layer). Inner layer and the gyroid model array intersected while the shell subtracted the cylinder model array in Magics 24.0, and then, the shell with gyroid pores and inner layer with round holes were obtained. Finally, the porous bioceramic scaffold model was obtained by merging these two porous models (Fig. 1B). And then the metal mesh was designed based on the defect model, with mesh structure to increase the contact area between the mucosa and the bioceramic scaffold.

### Fabrication of bioceramic scaffold and metal mesh

Bioceramic scaffolds were fabricated by DLP-based printing and sintering (Fig. 1C). First, the printing slurry was prepared by mixing 45% bioceramic powders, 50% SP700 photosensitive acrylic resin and 5% BYK-111 dispersant by weight. Then, the porous model was sliced and imported into the DLP printer (Shaoxing Xunshi Technology Co., China). The bioceramic slurry was cured layer by layer via the ultraviolet light below the slurry tank. The exposure time of each layer was set between 1.8 and 2.2 s while the cure depth is about 60 μm (Supplementary Fig. S1), and the cured layers with a thickness of 50 μm were manufactured. The printed bodies were washed, dried at 60°C and finally sintered (at 1150°C for CSi-Mg6, 1240°C for α-TCP and 1160°C for β-TCP) with a heating rate of 2°C/min.

Titanium meshes were fabricated by an SLM printer (BLT-S200; Bright Laser Technologies, China; see Fig. 1D) because of its good mechanical properties and biocompatibility [36]. The laser scanning speed was set to 800 mm/s and the layer thickness was set as 500 μm. After printing, NB 380 M vacuum furnace was used to complete the thermal treatment of titanium meshes with a temperature of 800°C for 4 h.

### Porosity, morphology and mechanical analysis

The sizes of porous scaffolds before and after sintering were measured, and then, the *X*–*Y* shrinkage and *Z* shrinkage were calculated. The porosity refers to the ratio of the void volume to the total volume of the scaffold and was calculated using the equation (*n* = 6): porosity (%) = (1 − *m*/(*ρ* × *V*)) × 100%, where *m* represents the mass of the sample, *V* is the total volume and *ρ* is the theoretical density. The microstructure of the scaffolds was studied by scanning electron microscopy (SEM, SU8010, Hitachi, Japan). The compressive strength and modulus of the bioceramic scaffolds (ø5 mm × 8 mm, *n* = 6) were tested using a universal testing machine (Instron 5566, Germany) to characterize the different mechanical properties of β-TCP, α-TCP and CSi-Mg6. The compressive strength and modulus were calculated by the formula: *σ_c_* = *F*/*A*, *E *= *σ_c_*/*ε*, where *F* is the ultimate load, *A* is the area of the cross-section and *ε* is the strain computed by the displacement divided by the original length. Meanwhile, the ultimate bending load and the ultimate compressive load of the sintered bioceramic scaffolds (13.0 mm × 5.9 mm × 4.0 mm, *n* = 6) were measured. The crosshead speed was 0.5 mm/min.

### Biocompatibility evaluation *in vitro*

To evaluate the biocompatibility of bioceramic scaffolds, MC3T3-E1 cells were used *in vitro* cell experiments. MC3T3-E1 cells were cultured with a growth medium containing α-MEM supplemented with 10% (v/v) FBS and 1% (v/v) penicillin and streptomycin in an incubator with 5% CO_2_ and 95% humidified atmosphere at 37°C. Gyroid-based porous scaffolds (7 mm × 3.5 mm × 2.5 mm) sterilized with 75% alcohol, washed with PBS and placed in a 24-well plate for cell experiments.

Cell proliferation and cytotoxicity of scaffolds were studied by the CCK-8 test. Each scaffold was implanted with 60 000 cells, and the wells with only cells were as control. The specimens were incubated in 10% (v/v) CCK-8 solution for 2 h after culture for 1, 4 and 7 days, and then, the optical density (OD) at 450 nm was measured using an enzyme marker (Epoch, BioTek, USA). The viability analysis was conducted according to the product manual of CCK-8.

The cell adhesion and cell morphology on the scaffold were studied by F-actin and nuclear fluorescence staining and SEM observation. About 20 000 cells were implanted in each specimen. After 1 day of planting, F-actin and nuclear fluorescence staining were performed for 40 and 5 min, respectively. The samples were observed under an optical microscope (F165MC; Leica, Germany). At the same time, other specimens were pre-fixed with glutaraldehyde solution overnight, then fixed with osmic acid and dried in a critical point dryer (HCP-2, Hitachi, USA), and SEM observation was conducted with SU8010 (Hitachi, Japan).

### Degradation evaluation *in vitro*

The scaffolds were weighed (*m*_0_) and immersed in Tris buffer (pH: 7.4) with a solid/liquid ratio of 1.0 g/50 ml at 37°C. The buffer was replaced every 7 days. At 2 and 4 weeks, the supernatant (0.2 ml) of all groups was extracted for ICP. Then, the corresponding scaffolds (*n* = 6) were washed with anhydrous ethanol, dried for 12 h and weighed again (*m_t_*) to calculate the mass change of the scaffolds. The mass residual was expressed as mass residual (%) = *m_t_*/*m*_0_ × 100%. Eventually, the mechanical tests were carried out again at 2 and 4 weeks, and the scaffolds were observed by SEM after 4 weeks of immersion.

### Animal experiments

All animal operations and experiments were approved by the Experimental Animals Ethics Committee of Zhejiang University (ZJU2020-10-093). A total of 48 rabbits (New Zealand White Rabbits, body weight: ∼3.0 kg) were used and divided into four groups, including the blank (without bioceramic scaffold), β-TCP, α-TCP and CSi-Mg6 groups. The critical-sized large defect (∼13 mm in length) was created in the right mandible, and then, bioceramic scaffold and metal mesh were implanted (Fig. 1E). First, the rabbit was anesthetized by the injection of 3% sodium phenobarbital at 1.0 ml/kg. The operative area of the lower jaw was shaved, cleaned and disinfected before the surgery. The mandible was exposed after creating an incision along the lower margin of the right mandible. The metal mesh was used as a guide to locate the defect area, and then, a large-size defect was established using a split drill. Afterwards, the bioceramic scaffold was implanted into the defect, and then, the scaffold was fixed with a metal mesh and two miniature titanium nails. After douching the surgery site with physiological saline, the incision was sutured. The rabbits were placed in cages and allowed to move freely after the operation. Penicillin was intramuscularly injected for 3 days to prevent infection. At 4, 8 and 12 weeks after surgery, four rabbits in each group were sacrificed by overdose anesthesia to evaluate new bone formation and bioceramic scaffold degradation.

### X-ray, μCT and mechanical analysis

After being sacrificed at a specific time point, the right mandible specimens of rabbits were collected. All the specimens were fixed in 4% paraformaldehyde. X-ray scanning (XPERT, KUBTEC, USA) was then performed after removing the titanium mesh and titanium nails. After that, specimens were μCT scanned. The ratio of new bone volume to total volume (BV/TV), BV/TV distribution along the long axis of scaffolds, newly formed bone area to new bone volume (BS/BV), newly formed bone area to total volume (BS/TV), trabecular thickness (Tb.Th) and trabecular spacing (Tb.Sp) was calculated to assess the bone regeneration while the ratio of volume of residual scaffold to original scaffold (scaffold residual) was calculated to assess the degradation rate of β-TCP, α-TCP and CSi-Mg6. To calculate the BV/TV distribution, the scaffold was divided into two parts along the longitudinal axis: two ends of the scaffold (thereafter referred to as ‘edge’) and the center part of the scaffold (thereafter referred to as ‘center’). The length of each edge part is one-quarter of that of the scaffold while that of center part is one-half. And then BV/TV was calculated respectively. Besides, Tb.Th and Tb.Sp were calculated as described previously [37], while the value of BV, TV and BS was obtained via BoneJ in Fiji (NIH, USA) [38].

To study the mechanical property and mechanical stability of mandibular bone after implanting the scaffolds, compression tests were conducted using the previously mentioned mechanical test machine. The specimens (without metal mesh) fixed with 4% paraformaldehyde were trimmed to 20.0 mm × 10.0 mm × 4.0 mm, and compression tests were conducted along the long axis of the wet state specimens (*n* = 3).

### Histological evaluation

The specimens were dehydrated in a graded alcohol series (80–100%) and embedded in polymethyl-methacrylate. And then the specimens were sectioned horizontally at a thickness of 100–200 μm by a saw microtome (SP1600, Leica, Germany). Afterwards, the sections were ground and polished to 20–30 μm in thickness and stained with McNeal staining. Finally, the sections were observed under light microscopy (DMLA, Leica, Germany) at different magnifications to evaluate the area of new bone. The percentage of newly formed bone area to total area (BS/TS) was quantitatively evaluated using ImageJ.

### Statistical analysis

All the data presented above were expressed as the mean ± standard deviation and analyzed by non-repetitive two-factor analysis. For all of the statistical results, *P < 0.05 stands for statistically significant.

## Results

### Primary characterization of powers and scaffolds

**Figure 2.**
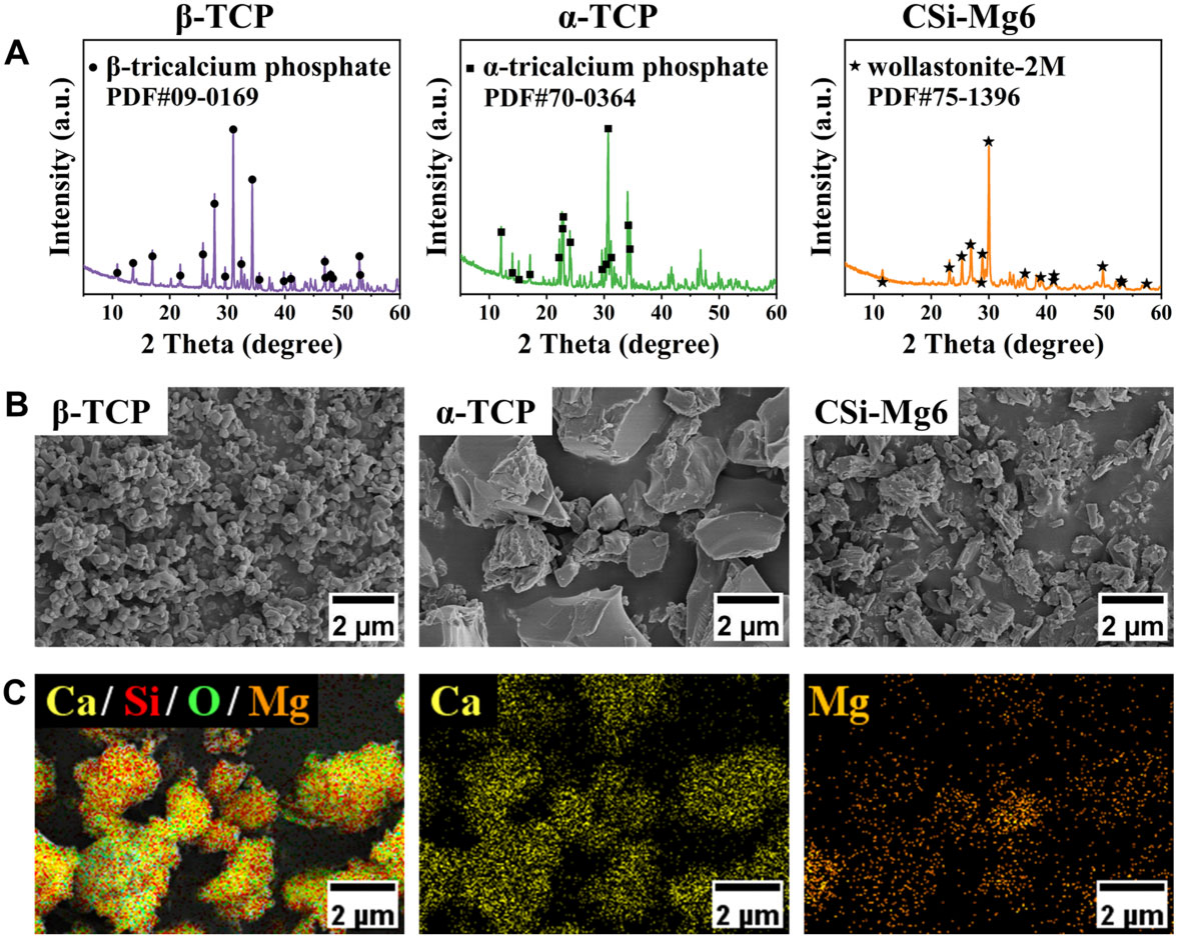
Primary characterizations of the powders. (**A**) XRD patterns of bioceramic powders, (**B**) SEM images of the powders and (**C**) elements distribution of CSi-Mg6 powders.

According to ICP analysis, the content of magnesium in CSi-Mg6 powder was 5.8 mol%, which is close to the designed value (6.0 mol%). XRD patterns (Fig. 2A) showed that the diffraction peaks of the β-TCP, α-TCP and CSi-Mg6 samples belonged to the β and α phases (PDF # 09-0169, 70-0364) of tricalcium phosphate and wollastonite-2M (PDF # 75-1396), respectively. Although the average particle size of the resulted powders was different, that of all powders was smaller than 5 μm, while the average particle size of CSi-Mg6 powders was medium (Fig. 2B). SEM-EDS result indicated that the distribution of Mg elements was relatively uniform (Fig. 2C).

**Figure 3.**
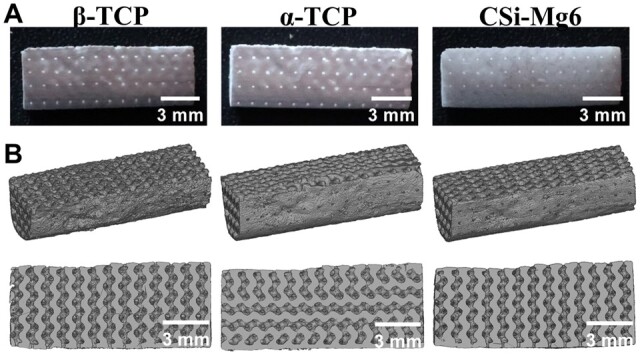
Images of the bioceramic scaffolds. (**A**) Images of the scaffolds and (**B**) μCT-reconstructed porous scaffolds.

**Figure 4.**
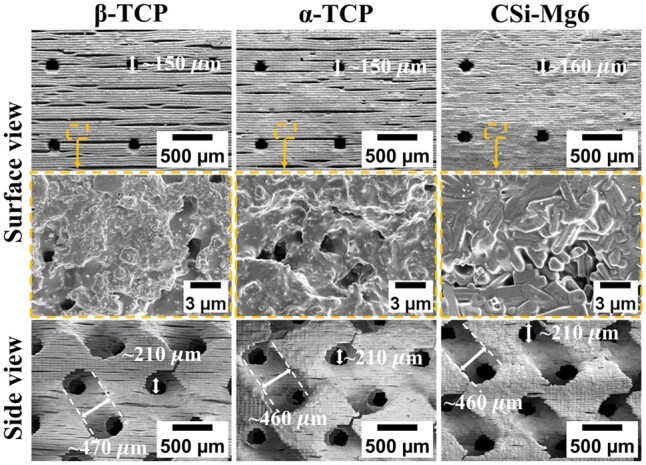
SEM images of the bioceramic scaffolds in surface view and side view. The down-row images of the surface view represent the magnification of the dotted boxes in the up-row images.

The representative external and internal macroporous structures of sintered scaffolds could be revealed by naked-eye observation and 2D/3D μCT reconstruction (Fig. 3A and B). The sintered bioceramic scaffolds retain the pore morphology of the 3D model and full pore connectivity. It could be seen that the bioceramic scaffolds showed different degrees of linear shrinkage (Table 1). The porosity of all sintered scaffolds was over 50%, but the CSi-Mg6 had the lowest porosity (51.2 ± 4.6%). The SEM images showed that there were evenly distributed macropores in the outer layer of scaffolds, and the internal gyroid pore geometry was maintained very well (Fig. 4). It was worth mentioning that there were obviously oriented microcracks on the surface of α-TCP and β-TCP scaffolds whereas there were few cracks on the CSi-Mg6 scaffolds. Moreover, the CSi-Mg6 scaffolds showed more appreciable densification in comparison with the TCP scaffolds.

**Table 1.** The shrinkage and porosity of porous bioceramic scaffolds (*n* = 6)

Sample	Sintering condition (°C)	*X*–*Y* shrinkage (%)	*Z* shrinkage (%)	Porosity (%)
CSi-Mg6	1150	29.60 ± 0.58	30.31 ± 0.44	51.39 ± 1.76
α-TCP	1240	18.69 ± 0.52	18.91 ± 1.41	63.99 ± 0.55
β-TCP	1160	25.31 ± 2.70	26.16 ± 2.37	56.06 ± 2.08

**Figure 5.**
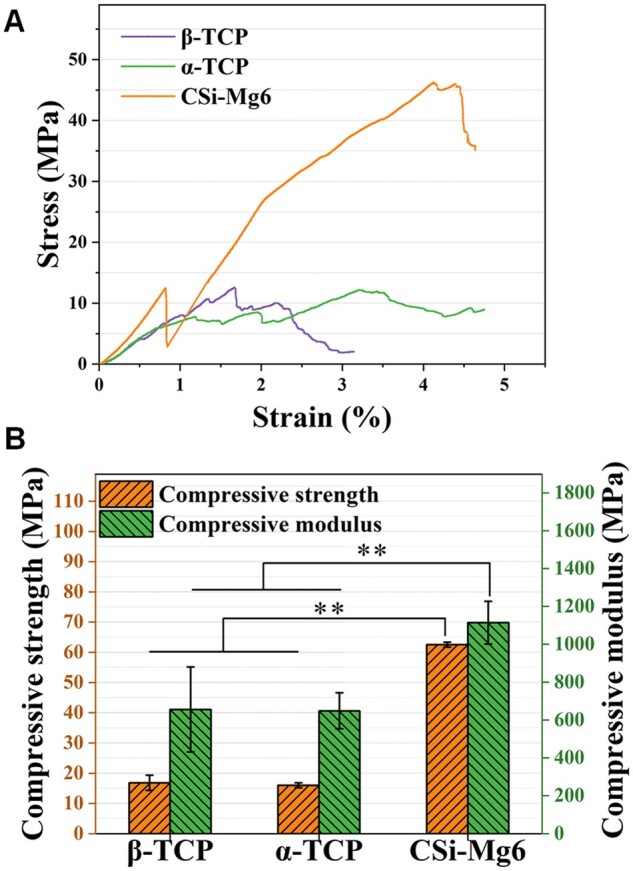
Mechanical characterization of the scaffolds. (**A**) Stress–strain curve of the scaffolds. (**B**) Compressive strength and modulus of the scaffolds (**P < 0.01).

To evaluate the structural stability of the bioceramic scaffolds *in vitro*, the compressive and bending tests were carried out on the sintered scaffolds. It was shown that the compressive and flexural load of CSi-Mg6 scaffolds were 3- to 4-fold higher than those of the TCP scaffolds (Fig. 5), suggesting the CSi-Mg6 scaffolds exhibited appreciable sintering property and mechanical resistance.

### Degradation testing *in vitro*

**Figure 6.**
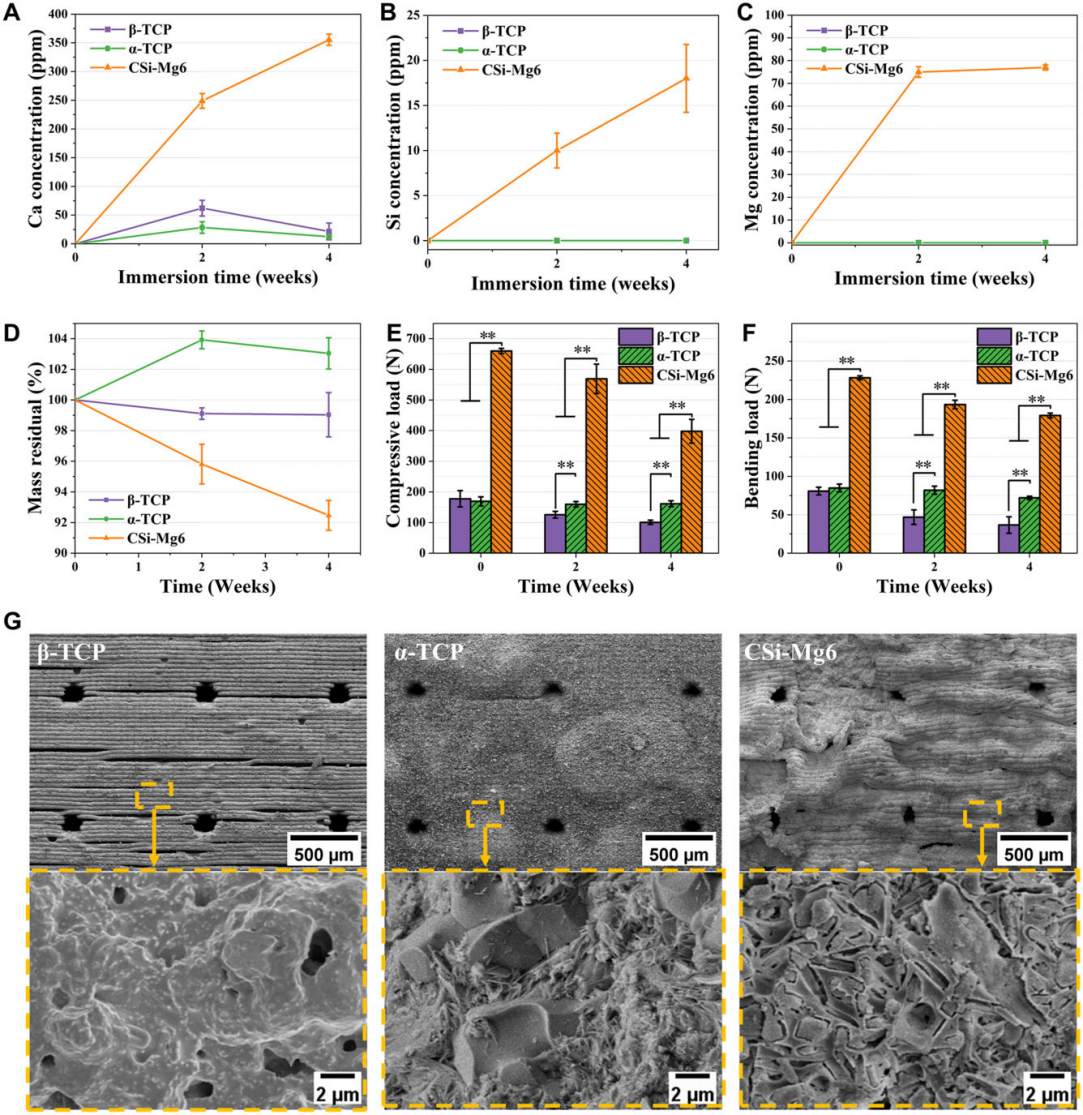
*In vitro* degradation characterization of the bioceramic scaffolds. (**A**–**C**) The ion release of Ca, Si, and Mg of the scaffolds. (**D**–**F**) The mass residual, the ultimate compressive load, and the ultimate bending load of scaffolds before and after immersion in Tris buffer. (**G**) The SEM observation of scaffolds after immersion in Tris buffer for 4 weeks (**P < 0.01).

It could be seen from Fig. 6A that the calcium ion concentration was much higher from the CSi-Mg6 scaffolds than the others, and meanwhile, the calcium ion release of α-TCP and β-TCP scaffolds decreased slightly after 2 weeks. It was obvious that silicon ion concentration was increased for the CSi-Mg6 group during the whole immersion stage, while the magnesium ion concentration reached the plateau level (∼75 ppm) in Tris buffers after 2 weeks (Fig. 6B and C). It was interesting that these scaffolds showed significantly different mass decay in Tris buffer (Fig. 6D). The β-TCP scaffolds were sparingly dissolvable, and its mass decay was nearly ∼1% after 4 weeks. In contrast, the mass of α-TCP scaffolds was increased within the initial 2 weeks, which was possibly ascribed to the hydrolytic reaction and phasic transformation from TCP to HAp in the early stage [39]. After that, the mass of scaffolds declined slowly. As expected, the CSi-Mg6 scaffolds experienced more mass loss in the whole stage, and its mass residual was 95.82% and 92.47% at 2 and 4 weeks, respectively. As for the potential decay of mechanical strength, all three groups of bioceramic scaffolds showed a strength decrease during immersion in Tris buffer (Fig. 6E and F). The decay rate of final compressive and bending loads of CSi-Mg6 scaffolds at 4 weeks were 30.08% and 21.46%, respectively. In this aspect, the strength of CSi-Mg6 was still significantly higher than that of the other two types of TCP scaffolds. Additionally, the SEM observation showed that the surface of α-TCP scaffold became denser after immersing 4 weeks, while the surface of pore struts of CSi-Mg6 bioceramic scaffolds exhibited remarkably corrosive features mainly due to bio-dissolution (Fig. 6G).

### Biocompatibility assessment *in vitro*

**Figure 7.**
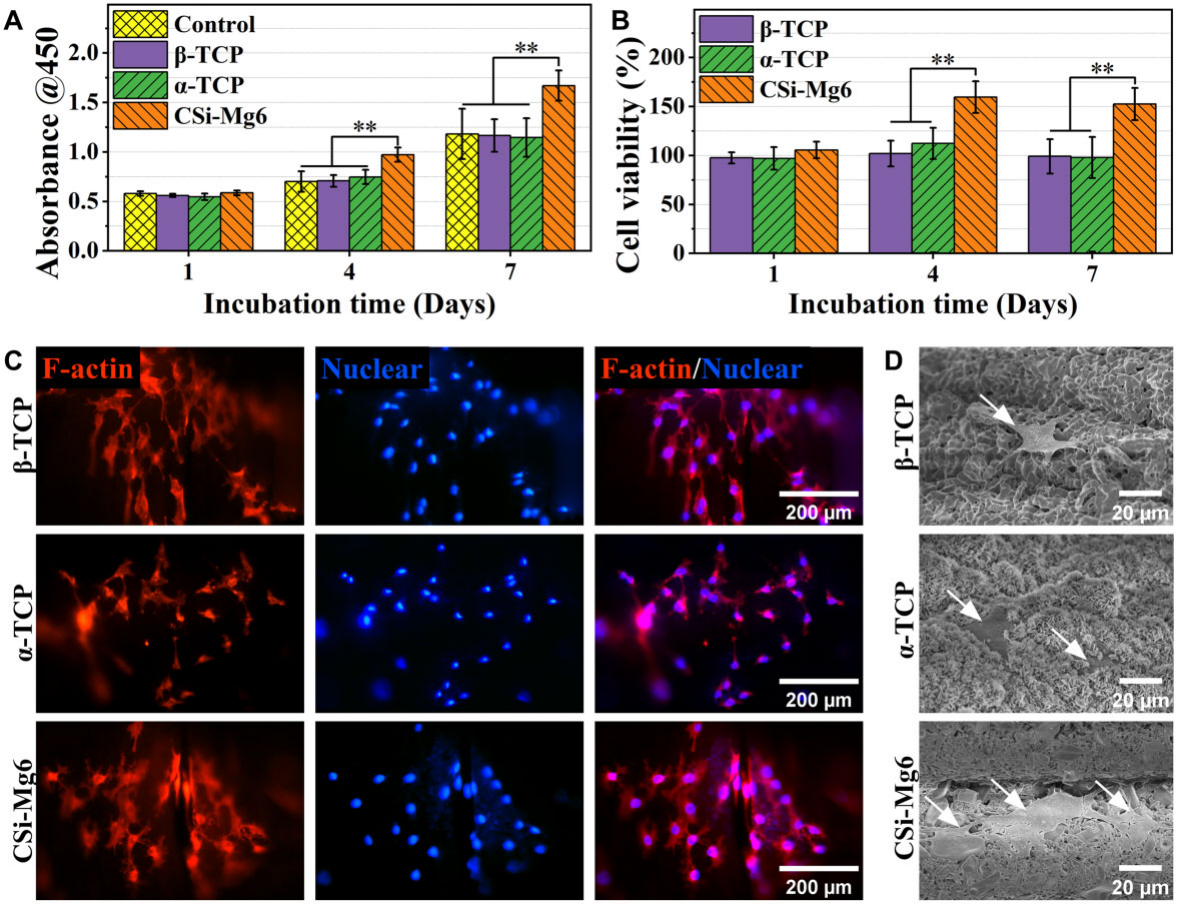
Biocompatibility of the bioceramic scaffolds. (**A**) Cell proliferation and (**B**) cell viability on the bioceramic scaffolds. (**C**) Fluorescence staining of cells. F-actin was labeled with phalloidin (red) while nuclei were labeled with DAPI (blue). (**D**) SEM images of cells on the bioceramic scaffolds. The white arrow indicated the cells (**P < 0.01).

According to the CCK-8 results, the OD values at 450 nm of the four groups were similar after 1-day incubation, while the CSi-Mg6 group showed higher OD values on Days 4 and 7 (Fig. 7A). The cell viability was calculated by comparison with the control group, that of TCP groups was around 100% during 7-day culture, indicating no cytotoxicity of β-TCP and α-TCP. Meanwhile, the cell viability of CSi-Mg6 exceeded that of the control group, indicating that CSi-Mg6 can promote cell proliferation (Fig. 7B). In the F-actin/nuclear fluorescence staining, the cells on the scaffolds all showed obvious filamentous pseudopods, which was also confirmed by SEM photos, especially the association between cells in the CSi-Mg6 group could be observed. We noted that the fluorescence intensity of the α-TCP group was weaker than that of the other two groups (Fig. 7C), and it might be explained by that the cells were covered by deposited apatite as shown in Fig. 7D.

### Macroscopic assessment *in vivo*

**Figure 8.**
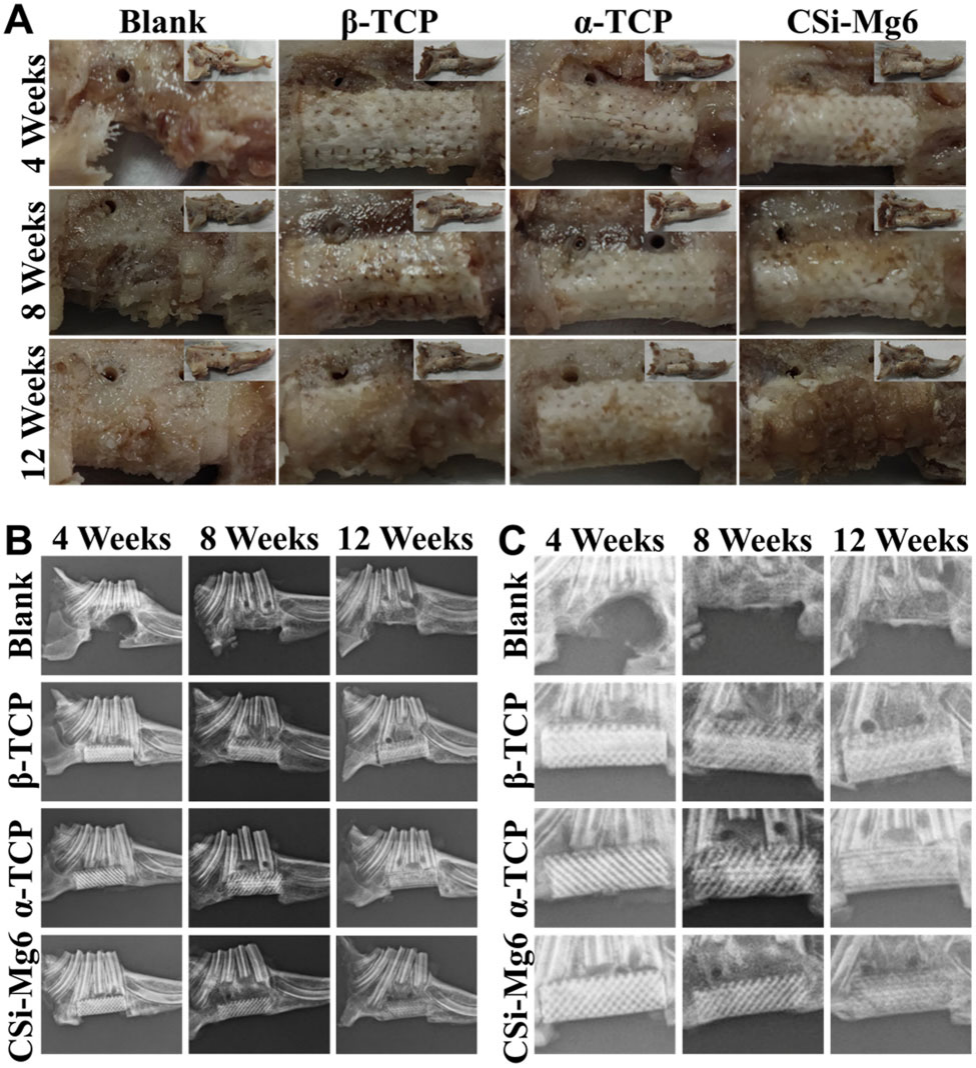
Gross observation and X-ray images of the specimens. (**A**) Gross observation of the specimens at 4, 8 and 12 weeks. (**B**) The overall X-ray images and (**C**) the enlarged X-ray images of the specimens at 4, 8 and 12 weeks.

To evaluate the osteogenic potential and bone repair efficacy of bioceramic scaffolds, *in vivo* osteogenic experiments were conducted by implanting the scaffolds into the large mandibular bone defect in rabbits. No animal death or obvious signs of infection were observed during the experiment. A gross examination of specimens showed that the scaffolds were firmly fixed by the titanium meshes and titanium nails (Fig. 8A). There was no necrosis or obvious inflammation in the mandibular specimens. According to the X-ray radiographic images, the defect was retained in the blank group while the boundary between scaffolds and host bone could be distinguished distinctly at 4 weeks (Fig. 8B and C). As for the specimens at 8 and 12 weeks, the material-tissue boundaries in the CSi-Mg6 and α-TCP groups became blurred at both ends of the long axis of scaffolds, and obviously, it implied these bioceramics possessed different mineralization appositional rates postoperatively.

### μCT examination *in vivo*

**Figure 9.**
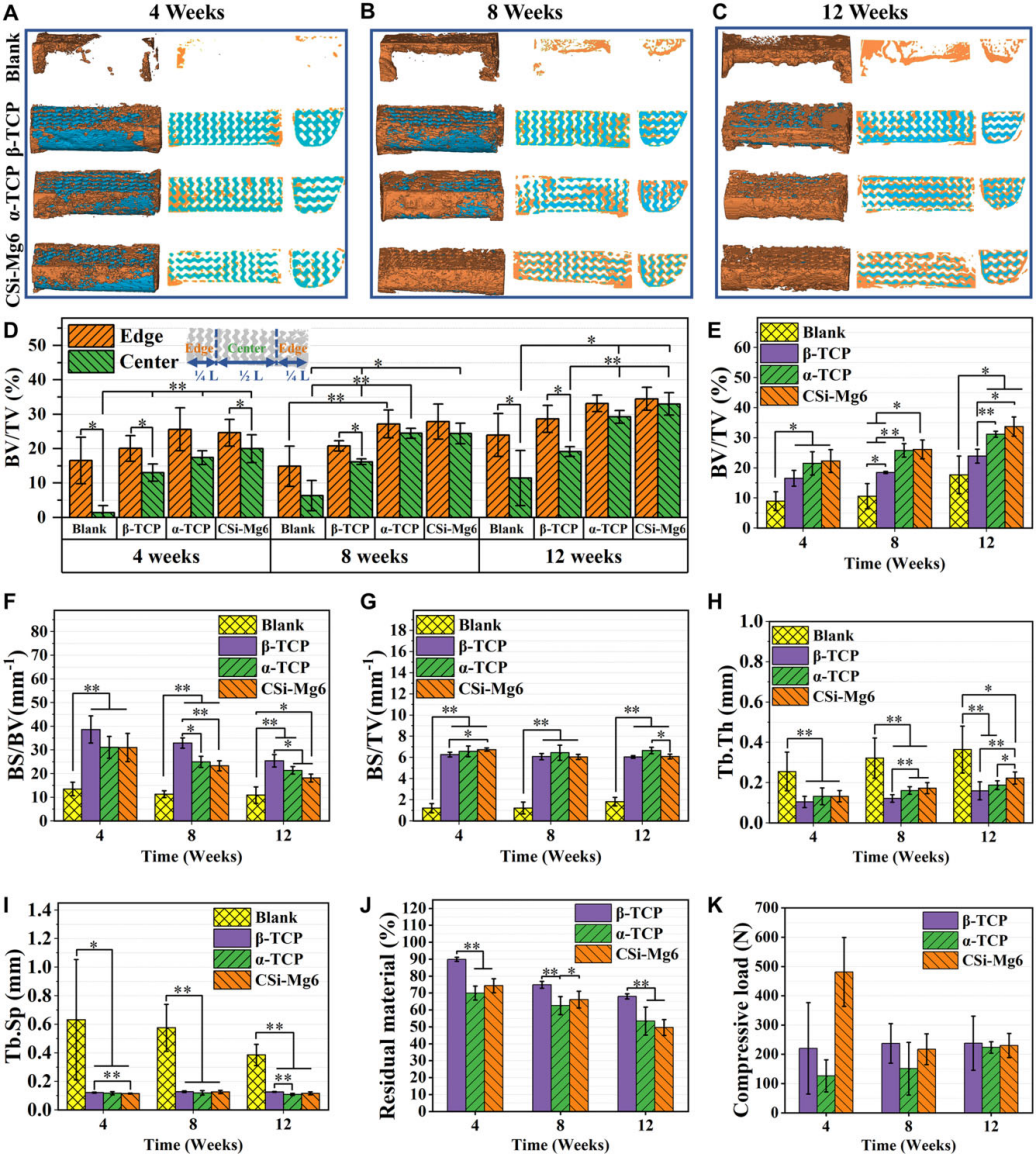
μCT reconstruction and quantitative analyses. (**A**–**C**) μCT reconstruction of new bone and bioceramic scaffolds at 4, 8 and 12 weeks, respectively. (**D**) BV/TV distribution along the long axis of the specimens. L: the length of the scaffolds. (**E**) BV/TV, (**F**) BS/BV, (**G**) BS/TV, (**H**) Tb.Th and (**I**) Tb.Sp analysis of the specimens. (**J**) Scaffolds residual analysis and (**K**) ultimate compressive load analysis of the specimens (*P < 0.05, **P < 0.01).

μCT measurements were taken to analyze the new bone formation and scaffold degradation. From the whole and transverse sections of reconstructed bioceramic scaffolds, all scaffolds were structurally integrated, and the new bone tissue (orange areas) gradually grew into the macropores of the scaffolds (blue areas; Fig. 9). A small amount of new bone formation was observed around the CSi-Mg6 and α-TCP scaffolds while very limited new bone tissue was present around the β-TCP scaffolds at 4 weeks (Fig. 9A). With the prolongation of repair time up to 8 weeks, the amount of new bone tissue was increased (Fig. 9B). The new bone tissue in the CSi-Mg6 and α-TCP groups was obviously higher and the new bone grew inward along the pores of the scaffolds. In contrast, new bone in the β-TCP groups remained at the interface between the scaffolds and host bone. After 12 weeks, all three groups showed a certain amount of new bone formation (Fig. 9C). However, the CSi-Mg6 and α-TCP groups exhibited more osteogenesis compared to the β-TCP and blank groups.

To further analyze the changes in the new bone tissue formation, BV/TV and BV/TV distribution along the long axis of scaffold was calculated (Fig. 9D and E). The results showed that more new bone grew at the edges than at the center among all groups at 4 weeks, indicating that all groups were repaired from the ends toward center of the scaffold. Interestingly, there was no significant difference in the volume of new bone at the edge of the defect, whereas there was a significant difference in new bone formation at the center of the defect at 4 weeks (*P* < 0.01). The CSi-Mg6 group had the best performance, followed by the α-TCP group, while the blank group had the least new bone formation. Both the CSi-Mg6 and α-TCP groups began to have a relatively balanced new bone distribution at 8 weeks, while the distribution differences of the β-TCP and blank groups still existed at 12 weeks. Hence, the blank and β-TCP groups displayed a limited increase in the value of total BV/TV during the whole implantation period while the CSi-Mg6 group and the α-TCP group showed excellent capacity of osteogenesis. The value of overall BV/TV of the blank group was much lower than that of the scaffold groups in 4 and 8 weeks, but there was no significant difference between the blank and β-TCP groups after 12 weeks. As for BS/BV, there was a significant difference between the blank group and the scaffold groups during the whole period and there was a relatively small difference between the scaffold groups (Fig. 9F). The β-TCP group had the largest BS/BV, followed by the CSi-Mg6 group and the α-TCP group, and that of the blank group was the smallest. Although BS/BV of all groups decreased over time, the order remained unchanged. Similar to BS/BV, the BS/TV of the scaffold groups was also much larger than that of the blank group (Fig. 9G). As for the quantitative measurement of trabecular morphology, we found that the thickness of bone trabecular increased over time, and that of CSi-Mg6 was the largest in the scaffold groups at 12 weeks, followed by the α-TCP group (Fig. 9H and I). However, the blank group had higher Tb.Th and Tb.Sp during the whole process. Although Tb.Sp of the blank group showed a decline during the whole process, it was still much higher than the scaffold group at 12 weeks. The results showed that the residual material (%) of the β-TCP group remains the highest, whereas the α-TCP and CSi-Mg6 groups showed much less scaffold residual during the whole implantation period, proving that α-TCP and CSi-Mg6 have faster degradation rate (Fig. 9J).

### Mechanical evaluation of specimens

Compression testing was conducted to evaluate the effects of different scaffolds on the mechanical property of the mandible. Maximum compressive forces instead of maximum compressive stresses were shown because of the irregular shape of the mandible and uneven bone trabecular structure (Fig. 9K). The results showed that the mean maximum compressive force of CSi-Mg6 specimens was about 481 N after 4 weeks of implantation, while that of the β-TCP specimens was less than half of that and the ultimate compression load of the α-TCP group is the lowest. At 8 weeks, the mechanical capacity of CSi-Mg6 specimens decreased significantly compared with that of 4-week samples. The mechanical property of the β-TCP group was basically unchanged while that of the α-TCP group improved slightly, but the α-TCP group was still the worst. At 12 weeks, the maximum compression load of the CSi-Mg6 and β-TCP groups remained stable, while the mechanical properties of the α-TCP group increased and the ultimate forces of the three groups were similar.

### Histological analysis

**Figure 10.**
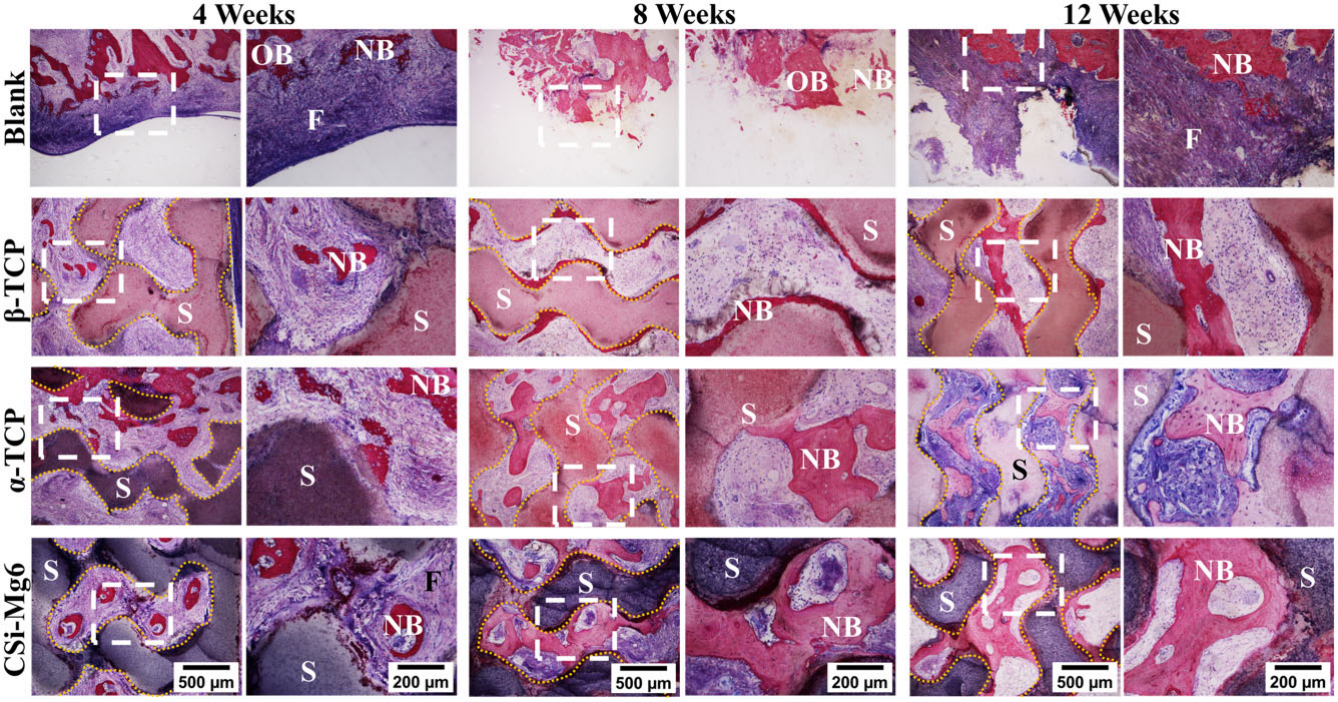
Histological analysis of McNeal-stained specimens at 4–12 weeks postoperatively. NB: newly formed bone; OB: original maturing bone; S: scaffold; F: fibrous tissue. The yellow dotted line outlines the scaffolds.

**Figure 11.**
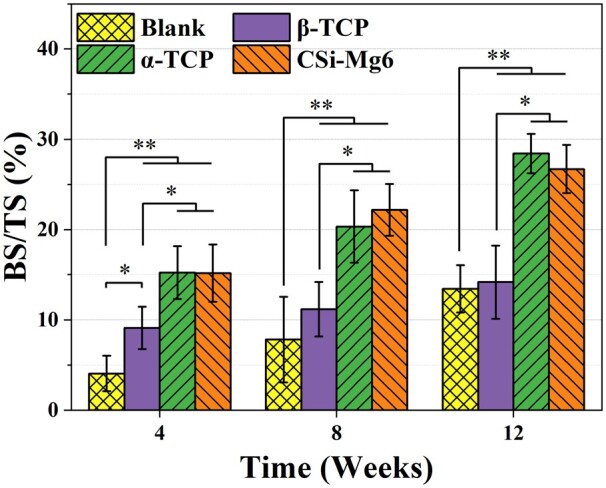
BS/TS analyses of McNeal-stained specimens (*P < 0.05; **P < 0.01).

The histological staining was performed to investigate bone tissue healing in mandibular bone defects with different scaffolds implanted (Fig. 10). The McNeal staining images of the specimens showed that the extent of new bone ingrowth was different within different materials. There was no obvious necrosis or inflammation in each group. The blank group retained the defect and could not heal spontaneously. New bone mainly formed close to the bioceramic scaffolds and extended into the interconnected pores with the passage of time. At 4 weeks, a small amount of new bone was formed adjacent to CSi-Mg6 and α-TCP struts, while new bone only formed where the scaffolds contact the host bone in the β-TCP group. At 8 weeks, all three groups showed an increase in bone formation, but the difference in the new bone area was significant, with the new bone formation of the CSi-Mg6 group higher than that of the other two groups. After 12 weeks, the α-TCP group showed significant biodegradation while the CSi-Mg6 group also had certain degradation phenomenon since the edges of the scaffolds were gradually replaced by new bone tissue. Both the CSi-Mg6 and α-TCP groups showed considerable osteogenic capacity, with plenty of mature bone evenly distributed in the interconnecting macropores, compared to the β-TCP group of that the proportion of new bone was much lower. In addition, the BS/TS result confirmed that the CSi-Mg6 and α-TCP groups had superior osteogenic ability than the β-TCP group (Fig. 11).

## Discussion

It is well known that nowadays, the repair of large-size load-bearing bone defect remains a challenge. Porous materials are required to be bioactive and biodegradable for *in situ* bone regeneration and bone defect repair [40]. In this respect, bioceramics have unique advantages because of their similar composition to bone [41]. Reconstruction of bone defects involves in the complex process of bone regeneration, and the scaffolds should not only match the bone defect well which is beneficial to the early stabilization and osseointegration between scaffolds and host bone but also possess microstructure to provide channels for tissue ingrowth, regeneration, nutrition and metabolism [42–44].

In this study, μCT scanning and 3D reconstruction techniques were used to obtain porous scaffold matching the profile of bone defect while DLP printing was used in the manufacture of fully interconnecting porous scaffolds. At the same time, the porous scaffolds should be firmly fixed to the host bone and have sufficient structural stability. Hence, it can undergo the local host pressure and provides temporary mechanical support for new tissue ingrowth. On one hand, although the CSi-Mg6 has achieved quite high compressive and flexural strength, its brittleness nature is challengeable for repairing the load-bearing bone defects [45]. Therefore, the metal mesh was designed and used to ensure the fixation and load bearing. On the other hand, our studies found that the combination of CSi-Mg6 scaffold and metal mesh is a promising strategy for large-size bearing bone repair, for the excellent osteogenesis, appropriate degradation rate and good structural stability are provided by CSi-Mg6 scaffold, while the firm fixation and stable mechanical bearing capacity are provided by the metal mesh.

To achieve clinical applications of bioceramic scaffolds, it is necessary to experiment with animal models of large (critical-size) bone defects. An 11-mm full-thickness defects have been identified as a critical-sized defect in the rabbit mandible [46]. In the present study, the mandibular large-size defect was designed with the help of digital technology. The defect was about 13 mm in length, which exceeded the established critical size. X-ray observations and 3D reconstruction of μCT scanning revealed thatthe defects were not fully filled with new bone, thus forming discontinuous mandibular lines in the blank group, although comparable new bone volume was achieved in this group at 12 weeks (Figs 8B and 9C and E). More cortical bone formed along the defect margin in the blank group, which is confirmed by the smaller value of BS/BV (Fig. 9F). The cortical bone had lower porosity than cancellous bone, which explained why the defect did not repair when the BV/TV almost reached one fifth. Interestingly, the histological staining further proved that the new bone tissue in the blank group was mainly distributed in the peripheral region of bone defects and fibrous tissues occupied the defect cavity, while the new bone tissue in the bioceramic scaffold group showed spatial distribution characteristics (Fig. 10). These results indicate that the bioceramic scaffolds provide the porous architectures favorable for new bone tissue ingrowth, and meanwhile, the small pores on the surface layer of the scaffold could readily prevent the invasion of soft tissue which may ensure the continuous ingrowth of new bone tissue. That is, it is proved that our animal model is valuable for evaluating the large-size bone defects in which the defect could not heal by itself as the blank group.

It is well established that the structure and porosity of scaffolds are connected with bone regeneration and repair [43]. TPMS-typed pore geometry can not only mimic the pore morphology of natural trabecular mineral networks and the pore parameters including pore size and pore interconnectivity but also has been proved to have ideal new bone growth *in vivo* [18, 19, 47]. Hence, TPMS-based gyroid architecture was chosen as the macropore architecture. However, the gyroid structure imposes higher requirements on the manufacturing process. DLP technique is an advanced manufacturing technique with a high resolution whose accuracy can be less than 100 μm, which was used in our study to produce porous scaffolds. The scaffolds with a specific shape and fully interconnected porous structure were successfully fabricated by DLP printing (Fig. 3B). Film should be used to prevent the growth of soft tissues after the implantation of osteogenic materials in the clinic [48]. And in our study, the shell with small pores was used instead of the film. As shown in gross observation and X-ray images (Fig. 8), malunion occurred in the blank group. The formation of new bone only around the defect and the large amount of fibrous tissue in the defect indicate that malunion is related to soft tissue invasion (Figs 9C and 10). However, this situation did not occur in the scaffold groups. Hence it might be inferred that our design played a role in preventing soft tissue invasion. In addition, 3D reconstruction of mandible samples further elucidated that the general shape of bioceramic scaffolds perfectly matched the mandibular defects, which means that the high-precision DLP printing technology is able to produce the personalized scaffolds (Fig. 9A–C).

Besides the pore structure of bioceramic scaffolds, the adequate mechanical stability is especially required [12]. The animal’s chewing function needs to maintain after the operation, which was predicted to promote bone repair. The constant physiological dynamic mechanical stimulus was beneficial for promoting osteogenic stem cell differentiation and, in turn, enhancing osteogenesis and angiogenesis [49, 50]. Therefore, the implant requires firm fixation and structural stability, and in this aspect, the metal mesh can contribute to the supporting effect on the porous scaffolds. General observation showed that there was no dislocation in the bone defects of the scaffolds throughout the experiment (Fig. 8A). The X-ray results showed that the bioceramic scaffold was tightly bound to the host bone tissue (Fig. 8B and C). These results proved that the titanium meshes play an important role in maintaining the integration of the scaffold and host bone tissue. Indeed, the bioceramic scaffolds still require structural stability to ensure the new bone ingrowth in the porous architecture of scaffolds. In fact, the long-term bending and compressive resistance of the CSi-Mg6 scaffolds in an aqueous medium were much higher than the other TCP scaffolds (Fig. 6E and F). The mechanical strength of the CSi-Mg6 scaffolds was still 2- to 5-fold of TCP scaffolds. The mechanical property of the *in vivo* specimens showed that CSi-Mg6 specimens had superior mechanical capacity compared with β-TCP and α-TCP specimens at the early stage of implantation (4 weeks), while α-TCP specimens had the worst mechanical performance (Fig. 9K). After 8 weeks of implantation, the mechanical strength of CSi-Mg6 specimens decreased, which was due to that mechanical attenuation caused by scaffold degradation overweighted mechanical strengthening caused by new bone formation. At 12 weeks, the ultimate compressive load of CSi-Mg6 specimens was equivalent to that of 8-week implantation, indicating that the balance was achieved. Interestingly, the maximum compressive force of α-TCP specimens showed the opposite trend. Although both α-TCP and CSi-Mg6 scaffolds degraded relatively fast, different initial mechanical properties led to various ultimate load changes, that is, the lower initial mechanical capacity of α-TCP led to the dominant mechanical strengthening due to osteogenesis (Figs 5B and 9J). The ultimate compressive load of β-TCP samples remained stable, which is an ideal property, but at the cost of a slower degradation rate. Then, the new bone volume was significantly smaller than that of the α-TCP and CSi-Mg6 groups at 12 weeks. Although none of the scaffolds collapsed during the observation period with the help of the titanium mesh, CSi-Mg6 scaffolds clearly provided additional protection during the early implantation period. These results demonstrated that the CSi-Mg6 scaffolds have appreciable mechanical properties and structural stability in comparison with the TCP bioceramics during the early stage of implantation.

On the other hand, the biodegradation of scaffolds is also needed to consider in the design of scaffolds, because the scaffold residual would take up the space and hinder new bone formation [51]. *In vitro* biodegradation experiments showed that the CSi-Mg6 scaffold had the fastest degradation rate, followed by β-TCP, while the mass of α-TCP was increased (Fig. 6D) due to the hydrolysis reaction and phase transformation [39]. This can also be verified from the SEM observation, in which only β-TCP scaffolds retained the micro-cracks after soaking (Figs 4 and 6G). *In vivo* results revealed that the biodegradation rate of CSi-Mg6 scaffolds was similar to that of α-TCP scaffolds, but the biodegradation rate of β-TCP scaffolds was much lower than the others (Fig. 9J). This may be explained that the *in vitro* biodegradation only produces a bio-dissolution process and fails to reflect the cell-mediated biodegradation mechanism [45]. In line with the biodegradation rate, the CSi-Mg6 scaffolds had significantly higher ion release than the TCP counterparts (Fig. 6A–C). The calcium ions will participate in the deposition of new bone tissue, and silicon and magnesium ions can also promote bone formation [52–54]. It can be inferred that the release of these inorganic ions would benefit the bone repair efficiency of the CSi-Mg6 scaffolds.

Moreover, our study reveals that the CSi-Mg6 and α-TCP groups have superior osteogenic ability. The μCT reconstruction showed that the CSi-Mg6 and α-TCP groups achieved new bone bridging at 12 weeks (Fig. 9C). Meanwhile, the quantitative analysis showed that the new bone volume in these two groups at 4 weeks was similar to that of the β-TCP group and the blank group at 12 weeks (Fig. 9E). Ito et al. also found that the osteogenic ability of β-TCP is not satisfying enough. The new bone volume formed in the β-TCP scaffolds in the alveolar fracture model was only half of that of autologous scaffolds [55]. In our study, the osteogenic capability of biomaterials is related to the release of bioactive ions during biodegradation. The rapid biodegradation of CSi-Mg6 and α-TCP scaffolds provided a large amount of calcium, silicon or phosphorus ions, contributing to the promotion of new bone deposition. The magnesium ion release and higher cell viability of the CSi-Mg6 scaffold can also contribute to the larger trabecular thickness at 12 weeks than in the α-TCP group (Figs 7B and 9H). Notably, the volume of new bone in the β-TCP group was significantly higher than that in the blank group at 4 and 8 weeks, while the amount of new bone was similar at 12 weeks, indicating that the slow biodegradation may retard the new bone formation. Moreover, the difference in osteogenic capability may also lie in the difference in the bone integration potential of biomaterials. The X-ray images showed that the interface between the ends of scaffold and host bone tissue was blurred at 8 weeks for the CSi-Mg6 and α-TCP groups, revealing the good bone integration of these scaffolds, but the β-TCP scaffold showed junior bone tissue integration until 12 weeks (Fig. 8B and C).

Finally, it is reasonable to consider that the combination of bioceramic scaffolds and metal mesh is a competitive strategy for the reconstruction of large-size load-bearing bone defects, and in particular, the personalized bioceramic scaffolds can be modeled by image scanning technology and printed by DLP high-precision printing technology. These investigations indicate that the CSi-Mg6 and α-TCP scaffolds exhibited more appreciable osteogenic capability and similar biodegradation rate *in vivo*; however, the former was superior in mechanical properties and long-term structural stability. It is assumed that CSi-Mg6 is more promising as biomaterials for large-range bone reconstruction in mandible defect conditions. Moreover, a longer time frame should be extended to determine when the whole bioceramic scaffold can be resorbed *in vivo*, and further studies are also needed to develop the personalized degradable mesh and staples to avoid secondary surgical removal.

## Conclusion

In summary, the 3D reconstruction technology was used to design bioceramic scaffolds with specific external morphologies and internal porous microstructures. The TCP and CSi-Mg6 scaffolds with appreciable sizes of and over 50% porosity were fabricated by DLP technology and investigated in mandible defect conditions. The 3D printed titanium meshes can play a good role in fixation requirement, which may help in resolving large bone defect regeneration and repair. Both CSi-Mg6 and α-TCP scaffolds have similar osteogenic performance and biodegradation rate *in vivo* while the latter showed low mechanical and structural stability. Hence, it is reasonable to consider that the CSi-Mg6 bioceramic scaffolds are more feasible for large load-bearing large-area mandibular bone defect repair in the future.

## Supplementary Material

rbad057_Supplementary_Data

## Data Availability

Data will be made available on request.
