# Supplementary material for: Customized bioceramic scaffolds and metal meshes for challenging large-size mandibular bone defect regeneration and repair
Source: Regen Biomater. 2023 Jun 7;10:rbad057. doi: 10.1093/rb/rbad057 (PMC10287912; doi:10.1093/rb/rbad057)
Supplement: rbad057_Supplementary_Data [file rbad057_supplementary_data.zip › Supplementary Figure 1.docx]

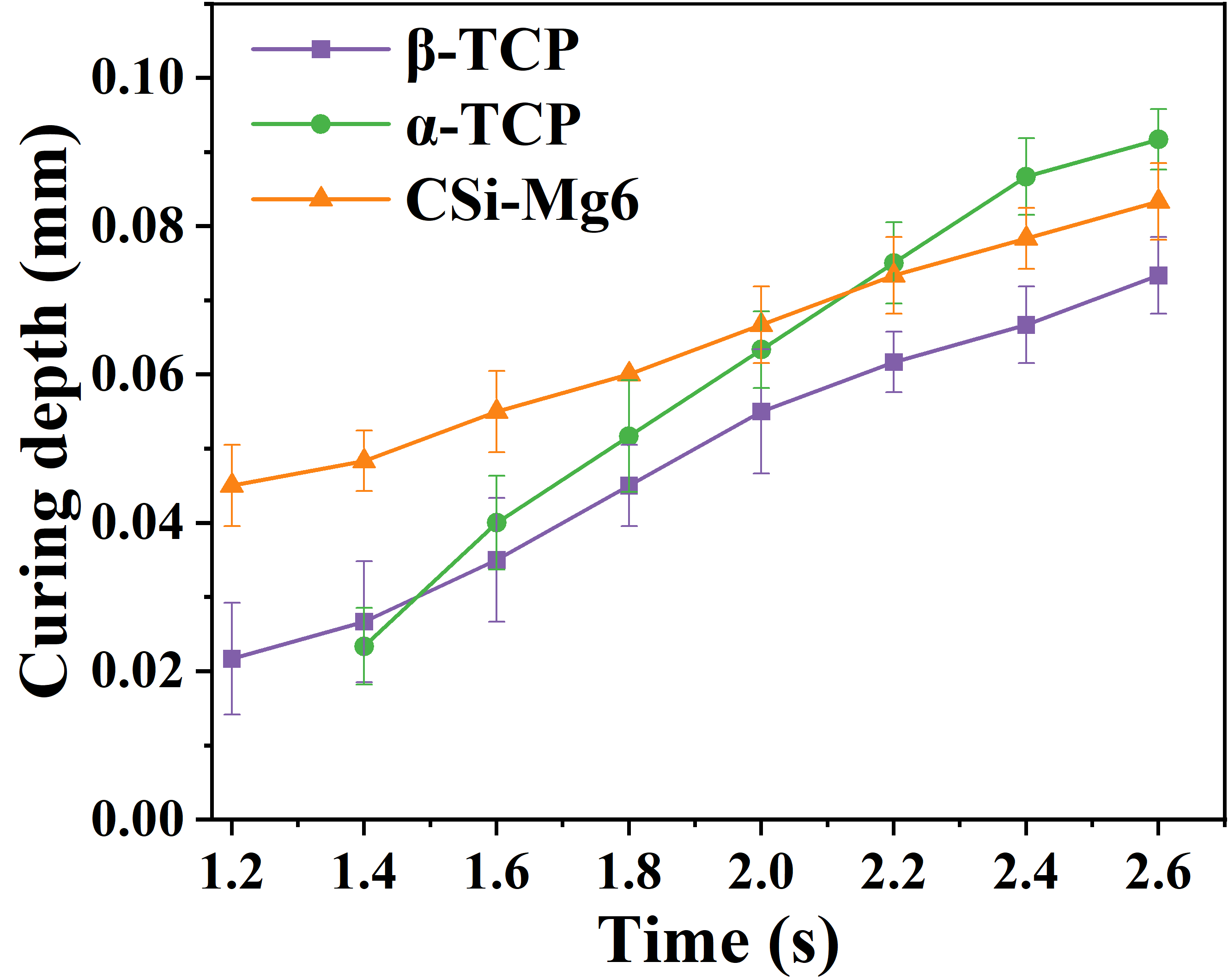


**Figure S1**. The relations between exposure time and curing depth. α-TCP did not cure when exposure time is 1.2 second.
