# Supplementary figures and images for: Customized bioceramic scaffolds and metal meshes for challenging large-size mandibular bone defect regeneration and repair
Source: Regen Biomater. 2023 Jun 7;10:rbad057. doi: 10.1093/rb/rbad057 (PMC10287912; doi:10.1093/rb/rbad057)

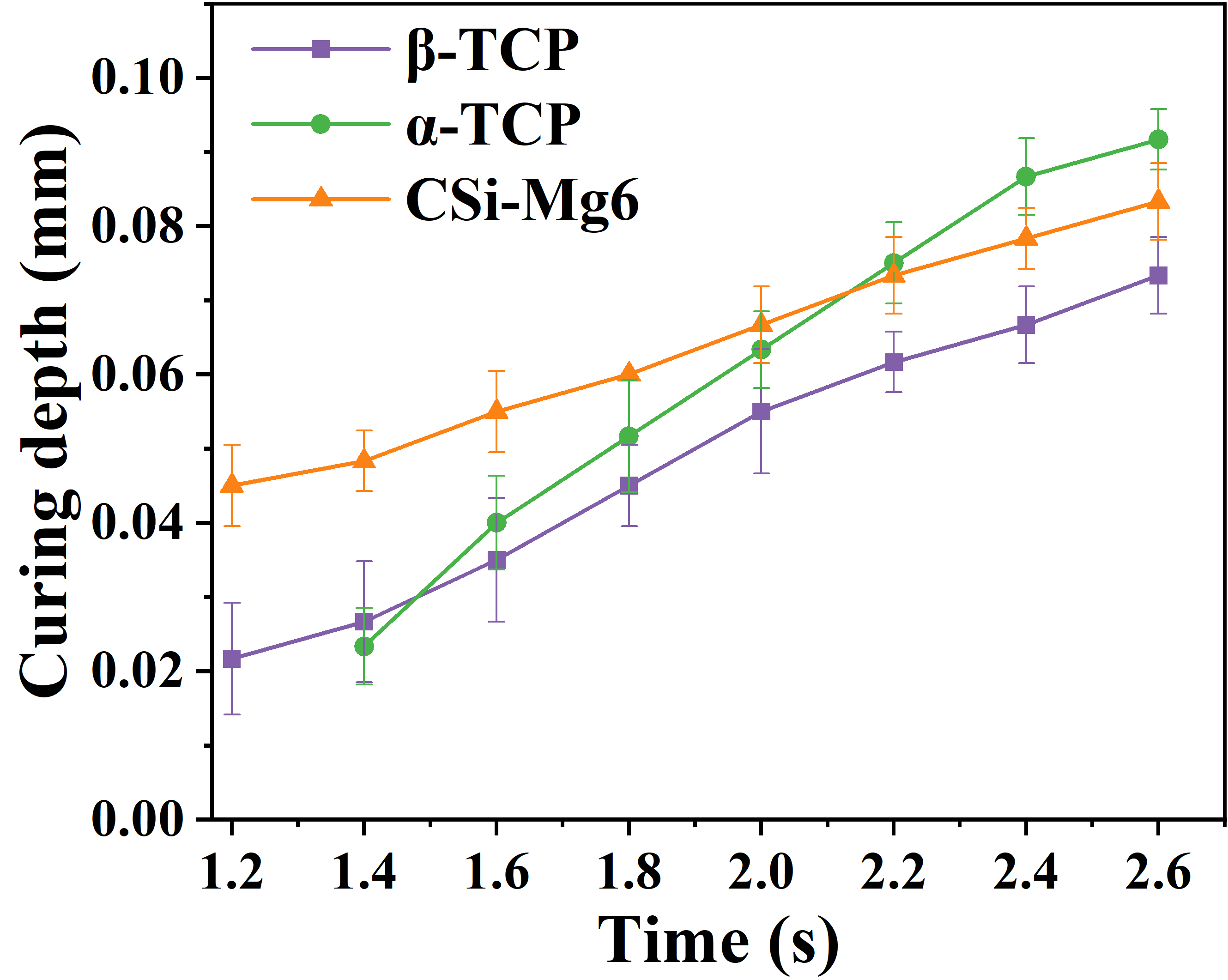

Supplement: rbad057_Supplementary_Data [file rbad057_supplementary_data.zip › OP-REGB230056_PECorr_AttachmentsFolder_Figure S1[AU].tif]
